# The Phorbol Ester Fraction from *Jatropha curcas* Seed Oil: Potential and Limits for Crop Protection against Insect Pests

**DOI:** 10.3390/ijms131216157

**Published:** 2012-11-30

**Authors:** Alain Ratnadass, Michael Wink

**Affiliations:** 1 Cirad, HortSys Research Unit, TA B-103/PS4, F-34398 Montpellier Cedex 5, France; 2 Institute of Pharmacy & Molecular Biotechnology (IPMB), Heidelberg University, Im Neuenheimer Feld 364, D-69120 Heidelberg, Germany; E-Mail: wink@uni-hd.de

**Keywords:** Physic nut, plant-derived insecticide, mode of action, field pests, crop pests

## Abstract

The physic nut shrub, *Jatropha curcas* (Euphorbiaceae), has been considered as a “miracle tree”, particularly as a source of alternate fuel. Various extracts of the plant have been reported to have insecticidal/acaricidal or molluscicidal/anthelminthic activities on vectors of medical or veterinary interest or on agricultural or non-agricultural pests. Among those extracts, the phorbol ester fraction from seed oil has been reported as a promising candidate for use as a plant-derived protectant of a variety of crops, from a range of pre-harvest and post-harvest insect pests. However, such extracts have not been widely used, despite the “boom” in the development of the crop in the tropics during recent years, and societal concerns about overuse of systemic chemical pesticides. There are many potential explanations to such a lack of use of Jatropha insecticidal extracts. On the one hand, the application of extracts potentially harmful to human health on stored food grain, might not be relevant. The problem of decomposition of phorbol esters and other compounds toxic to crop pests in the field needing further evaluation before such extracts can be widely used, may also be a partial explanation. High variability of phorbol ester content and hence of insecticidal activity among physic nut cultivars/ecotypes may be another. Phytotoxicity to crops may be further limitation. Apparent obstacles to a wider application of such extracts are the costs and problems involved with registration and legal approval. On the other hand, more studies should be conducted on molluscicidal activity on slugs and land snails which are major pests of crops, particularly in conservation agriculture systems. Further evaluation of toxicity to natural enemies of insect pests and studies on other beneficial insects such as pollinators are also needed.

## 1. Introduction

### 1.1. *Jatropha curcas* as a “Miracle Tree”

The physic nut shrub, *Jatropha curcas* (Euphorbiaceae), has been considered as a “miracle tree”, particularly as a source of alternate fuel, since seed oil can be used as a substitute to diesel oil [1–7]. Besides its production for this use (which “boomed” in recent years), the shrub is grown as live hedges to combat erosion, or as live fences to protect market gardens from domestic animals [8]. This adds to the more or less traditional use of the shrub for other purposes, particularly as medicines, in many parts of the world [9].

### 1.2. Biocidal Effects of *J. curcas* Extracts

Besides effect on human pathogens [10,11], various extracts of the plant have been reported to have insecticidal/acaricidal or molluscicidal/anthelminthic activities on vectors of medical or veterinary interest or on non-agricultural pests. These encompass mosquitoes [12–17], mites [18,19], cockroaches [20], houseflies [21], termites [22–25], and water snails hosts of human or cattle parasites (especially schistosomes) [26–34].

Toxicity of extracts of other species of Jatropha (e.g., *J. gossypifolia* or *J. podagrica*) has also been reported [35,36].

Among *J. curcas* extracts, the main biocidal action has been ascribed to the phorbol ester (tetracyclic diterpenoid) fraction [37,38] from seed oil. Six phorbol esters (*Jatropha* factors C1–C6) have been characterized from *J. curcas* seed oil with the molecular formula C_44_H_54_O_8_Na, as intra-molecular diesters of the same diterpene, 12-deoxy-16-hydroxyphorbol. Biocidal, including insecticidal activity of phorbol esters is due to the stimulation of the cellular target protein kinase C (PKC). Toxic phorbol esters are also found in other genera of the Euphorbiaceae family, particularly *Croton* and *Euphorbia*[39]. This fraction has also been reported as a promising candidate for use as a plant-derived protectant of a variety of crops, from a range of pre-harvest and post-harvest insect pests.

On the other hand, the physic nut tree is also subject to damage by several pests [40–43], but these insects are often specialists which are adapted to the toxicity of their host plant. Some specialists, such as the aposematic *Pachycoris klugii* (Hemiptera) may even sequester phorbol ester as chemical defense means against predators [44]. A few varieties of *J. curcas*, which have been selected by plant breeders, do not accumulate phorbol esters; the seed oil is no longer toxic to animals [37]. Seeds also contain a toxic lectin (a peptide) which however does not appear in the pressed oil [37].

The aim of this review is to take stock of all reported work on the insecticidal effect of physic nut (particularly *J. curcas*) and its extracts (particularly the oil and its phorbol ester fraction) on crop pests, and analyze the reasons for the lack of practical applications of the acquired knowledge, notably its translation into commercial products.

## 2. Insecticidal Activity of *J. curcas* Extracts (Particularly Phorbol Esters) on Pre- and Post-Harvest Crop Pests

### 2.1. Crops Studied

There are 10 crops on which *J. curcas* extracts have shown potential for protection against insect pests (Table 1).

Field crops encompass cotton [45,46], cowpea [47,48], maize [49], musk melon [50], okra [51], rice [50,52,53], and sorghum [54].

Stored crops encompass bean seeds [55], cowpea seeds [56], maize grain [57–59], mungbean seeds [46], potato tubers [60,61], rice grain [62,63], and wheat grain [64].

It should however be noted that not all studies involved relevant control/blanks to make sure that reported effects were due to the extracts, and not the solvents (e.g., [50], as opposed to [47]).

### 2.2. Crop Pests Studied

There are 37 insect pest species against which *J. curcas* extracts have been employed, and have shown biological activity (Table 2).

Coleoptera were the most represented, with 13 species in six families, encompassing mainly stored grain-feeding beetles [22,46,55–59,62–64,67–71,80], flea beetles [51,65,72] and a flower weevil [81].

Lepidoptera were the second most represented order, with 12 species in seven families, encompassing stem boring and leaf-feeding caterpillars [50,52,72,74,75,77–79], fruit or head-feeding caterpillars [45,46,49,50,54,66,72,76], and the potato tuber moth [60].

They were followed by sap-feeding piercing-sucking Hemiptera, with 11 species in five families [46–48,53,54,66,72,73,78]. Two studies showed activity on a flower thrips [47,48], and one a grasshopper species [82].

### 2.3. Types of Extracts and of Effects

Extracts that were studied and showed biological activity against insect pests of crops were mainly oil extracts, particularly phorbol esters (Table 3). Range of biocidal/biostatic pathways is quite broad (Table 3), encompassing contact toxicity on all developmental stages, ingestion toxicity, feeding deterrency and repellency (Table 3).

The most studied pest family has been that of Noctuidae, with 10 studies on 5 species (at par with Aphididae, but with only 4 studies on the latter). Noctuid caterpillars were either leaf feeding, stem boring, or fruit boring species, and were studied mainly in the laboratory, although some field tests on *Helicoverpa armigera* were conducted on cotton [22] and musk melon [50].

For *H. armigera* on cotton, synthetic insecticides were more effective than Jatropha oil (at the dose of 800 mL ha^−1^) at the start of treatment, as the oil affects only insect growth and its effect is therefore slower. On musk melon, a 70% reduction of infestation over the control was observed with a concentration of 0.5 mg mL^−1^ of methanol extract of Jatropha oil (dosage per ha not known).

As concentrations of phorbol esters in one of the studies on *H. armigera*[54] were actually mistakenly reported in g mL^−1^ instead of mg mL^−1^, in all laboratory studies, effective concentrations either for contact or ingestion deterrency/toxicity effect were consistently in the range between 0.1 and 1 mg mL^−1^ of phorbol esters (or their equivalent in oil).

However, one should be cautious concerning the efficiency of such extracts under field conditions, as translated by the little numbers of actual field tests. In addition, species with cryptic caterpillars like stem borers, particularly *Sesamia calamistis*, which does not have any wandering larval stage, and fruit worms (e.g., *Helicoverpa* spp.) are more difficult to control than leaf-feeding species. Foliar spraying targeting the latter (e.g., *Spodoptera frugiperda*) may on the other hand result in phytotoxicity on the crop (namely leaf burns), not necessarily due to phorbol esters *per se*, but to the oil or adjuvants in some formulations [22,47].

## 3. Lack of Development of Biocidal (Particularly Crop Pest Insecticidal) Applications of *J. curcas* Extracts and Potential Explanations

Since the first records of *Jatropha* oil biocidal activities on crop insect pests, almost thirty years ago, such extracts have not been widely used, despite the “boom” in the development of the crop in the tropics for biodiesel purpose during recent years, and increased public awareness of adverse environmental impacts of synthetic chemical insecticide use.

Phorbol esters activate PKC [39]. PKC is involved in several cellular signaling pathways. Some of them are active in cancer cells. If PKC is activated in cancer cells, their growth can be stimulated. Because of this property toxicologist classify phorbol esters as “co-carcinogens” or tumor-promoting. This term is somewhat misleading to the non-toxicologists, because it is easily mistaken for a carcinogen which can cause cancer. It should be remembered, that also estrogens, which are the active ingredients of anti-baby pills, have been classified as co-carcinogens because they stimulate growth of hormone-dependent cancer cells. The co-carcinogenic property of phorbol esters should not be an apriori “no-go” for a registration.

Such a lack of use was also reported for other plant-derived insecticides, deterrents, and repellents [83,84]. The main barriers to commercialization for botanical insecticides include: sustainability of the botanical resource, standardization of chemically complex extracts, and regulatory approval. The latter process is as cumbersome and expensive for a botanical insecticide as for a synthetic compound. Because it is difficult or even impossible to obtain patents for such phorbol ester extracts, agrochemical companies are not inclined to spend large amounts of money for toxicological tests and efficacy trials that are required for registration and approval as biopesticides.

### 3.1. High Variability of Phorbol Ester Content and Difficulty of Extraction

High variability of phorbol ester content and hence of insecticidal activity among physic nut cultivars/ecotypes [37] may be an explanation. This however can be solved by extraction/concentration, but this is not easy to achieve at the village level [75].

The analysis of phorbol esters requires sophisticated laboratory equipment such as HPLC and LC-MS [37,72], which is often lacking in tropical countries or in agricultural laboratories. Therefore, the selection of high-yielding varieties is difficult and labor-extensive. Furthermore, most emphasis in breeding of physic nuts was on the selection of varieties which were low on phorbol esters so that they could be used for animal or human consumption.

### 3.2. Non-Relevance of Application on Stored Grain

Another reason for the lack of development of pesticide uses might be the relevance of the application of such toxic extracts [39] on stored grain aimed at human consumption (Table 1). The fact that most studies refer to such post-harvest pests as *Callosobruchus maculatus* or *Sitophilus zeamais* (Table 2) is probably just an indication that these pests are easier to study than field pests, and that use of botanicals on stored produce is a traditional practice, more than on field crops.

On the other hand, human toxicity is mainly ascribed to the curcin protein which occurs in the seed meal but not in the oil. Seed oil also contains hydrogen cyanide. While phorbol esters, as co-carcinogenic (and not carcinogenic *per se*) compounds [39], are considered as less of a problem, although concentrated solutions are irritant to skin and eyes. Another problem could be toxicity of the extracts against germinating plants (phytotoxicity). At least, if cereal grains are used as seeds, their germination is not affected ruling out a phytotoxic effect of phorbol esters at this level [57].

### 3.3. Lack of Studies under Real Conditions

On both storage and field pests, studies were mainly conducted under laboratory conditions, so that potential field problems such as efficiency and persistence under natural conditions, phytotoxicity on crops (either of the oil, or of adjuvants in phorbol ester formulations [22,47]), and environmental fate, remain to be studied.

The problem of decomposition of phorbol esters and other toxic compounds in the field (from UV light and/or microbes) needs further evaluation before insecticidal or molluscicidal oil extracts can be widely used. This fact may actually also be a partial explanation to their lack of use. On the other hand, recent results showed that phorbol esters are biodegradable in the soil [85].

Further to the case of storage pests, this is another instance of conflicting effects between biological/biocidal activity against pests and persistence/innocuity for humans and the environment. Biodegradability and low persistence in the soil are a plus regarding human health and pollution, but a minus regarding bioactivity on soil pests (e.g., nematodes, on which there are several reports of bioactivity of Jatropha extracts, particularly oil seed cake [86–88]). A trade-off/balance has therefore to be found.

Regarding effects on non-target arthropods, low toxicity of extracts of other species of Jatropha was reported on beneficial insects or mites, namely predators [89,90], while conflicting results were obtained with *J. curcas* extracts on parasitoids [70]. On the other hand, in a field study, the better cotton yield with Jatropha oil treatment over farmer conventional practice was ascribed to the innocuity of oil for natural enemies [22].

In any case, further to studies on evaluation of toxicity to natural enemies of insect pests, such studies on other *beneficial* insects like pollinating insects and honeybees are still needed [91].

## 4. Conclusions

### 4.1. Prospects for the Development of *J. curcas* Extract Uses for Crop Protection

All the studies discussed indicate that extracts from *Jatropha* spp. (and particularly *J. curcas*) leaves, seeds and oil are repellent, deterrent or toxic, either by contact or ingestion, to several agricultural insect pests. The extracts involved mainly contain toxic diterpenes (particularly phorbol esters).

While the development of Jatropha cultivation for its mere insecticidal properties is unlikely, insecticidal compounds such as phorbol esters can be valuable by-products wherever the plant is grown as live-hedges or garden fences, or for biodiesel. As a biofuel (biodiesel), Jatropha has the highest potential in isolated or land-locked countries (e.g., small islands or Sahelian countries), where it has a clear comparative advantage over imported fossil fuels.

As mentioned above, in these contexts, phorbol ester extracts could be obtained as a rather cheap by-product of biodiesel production and would be available in developing countries which cannot afford to buy expensive agrochemicals. Furthermore, these nonrenewable chemical plant protection products are under threat of being banned anyway, due to their documented adverse effects on human health and the environment.

The extraction of the phorbol ester fraction of the seed oil of *J. curcas* using for example methanol as a solvent, does not affect its biodiesel properties [92], so that a small part of oil could be enriched with phorbol esters and used as a plant-derived insecticide. Such a “detoxification” could also help other uses, including as edible oil. Actually, such an “enrichment” would probably be the best trade-off [31], since these compounds are protected from heat when they are in oil, while their shelf-life is considerably reduced when they are extracted [93].

It should however be noted that such extraction/enrichment processes are not possible at the farmer level since it involves the use of solvents e.g. methanol, but could still be done at the village level at a semi-centralized scale (e.g., that of the village power plant) [75].

### 4.2. Potential for Combination of *Jatropha* Extracts with Other Control Options in Integrated Pest Management (IPM) or Agroecological Strategies

In view of its at least partial effects on crop pests, application of Jatropha oil or its phorbol ester fraction could be complementary with other control methods such as impregnated nets [94]. Actually, such a technique should be cost-effective on high-value crops such as vegetables or “concentrated” products such as or stored produce.

Furthermore, the reported antifungal properties of Jatropha extracts [95–101] could make it for the potential increased fungal infection risk due to microclimate alteration (higher humidity) under such nets.

Another option would be to combine the biocidal activity of Jatropha extracts and the use of trap plants, in a “push-pull” strategy, as we discussed earlier [54].

The phorbol ester fraction could also be combined with other botanical pesticides (see [83] for a review) that attack different molecular targets in pest organisms. Phorbol esters mainly activate PKC [72] and related signal transduction pathways. Their combination with a neurotoxin (e.g., nicotine, lupin alkaloids: [83]) might result in a synergistic efficacy. In synergistic combinations the dose of the individual component can be reduced, thus minimizing adverse effects, while delaying the emergence of resistant pest strains.

On the other hand, due to its documented toxicity on water snails hosts of human or cattle parasites [26–31], or agricultural pests [22], more studies should be conducted on molluscicidal activity on slugs and land snails which are major pests of crops in conservation agriculture [102], where risk of phytotoxicity and leaching is low, the product being applied on the (thick) mulch [103,104].

Broadly speaking, based on the existing literature and in view of the likely development of *Jatropha* cultivation in the tropics, the study of the insecticidal effects of its seed oil, particularly phorbol esters, and their evaluation in real conditions, should be given priority, rather than screening of novel active bioactive substances.

## Figures and Tables

**Table 1:** Crops on which *Jatropha curcas* extracts have shown potential as field or storage protectants.

Types of crops	Crop species	Field	Storage	Type of application	References
Cereals	Maize (*Zea mays*)	X		Oil emulsion	[49]
			X	Emulsifiable concentrate of seed oil; leaf extracts; seed oil	[46,57–59]
	Rice (*Oryza* sp.)	X		Seed oil: methanol, petroleum ether, ethyl acetate, benzene and water extracts; seed powder: acetone and aqueous extract; formulated seed oil	[50,52,53]
			X	Seed powder; seed powder acetone extract	[62,63]
	Sorghum (*Sorghum bicolor*)	X		Emulsifiable concentrate of seed oil methanol extract	[54]

Legumes	Bean (*Phaseolus aconitifolius*)		X	Seed oil	[55]
	Cowpea (*Vigna unguiculata*)	X		Emulsifiable concentrate of seed oil	[47,48]
			X	Seed oil	[56]
	Mungbean (*Vigna radiata*)		X	Emulsifiable concentrate of seed oil	[46]

Tuber	Potato (*Solanum tuberosum*)		X	Seed oil	[60,61]
Fruits/vegetables	Melon (*Cucumis melo*)	X		Seed oil: methanol, ethanol, petroleum ether, ethyl acetate, benzene and water extracts	[50]
	Okra (*Abelmoschus esculentus*)	X		Aqueous and petroleum ether extracts	[51,65]

Fibre	Cotton (*Gossypium* spp.)	X		Emulsifiable concentrate of seed oil	[45,46,66]

**Table 2:** Insect pest species which were affected by *Jatropha curcas* extracts.

Order	Family	Species	References
Coleoptera	Bostrychidae	*Rhyzopertha dominica*	[67]
	Bruchidae	*Callosobruchus chinensis*	[46]
		*Callosobruchus maculatus*	[55,56,68–70]
		*Phaedon cochliariae*	[71]
	Chrysomelidae	*Podagrica sjostedti*	[67]
		*Podagrica uniforma*	[67]
		*Podagrica* sp.	[51]
		*Sitophilus zeamais*	[46,57–59,62,63,67,69]
	Curculionidae	*Sitophilus granarius*	[64]
		*Amorphoidea lata*	[65]
	Silvanidae	*Oryzaephilus surimanensis*	[67]
	Tenebrionidae	*Tribolium confusum*	[68]
		*Tribolium castaneum*	[67]

Hemiptera	Aphididae	*Myzus persicae*	[72]
		*Lipaphis erysimi*	[73]
		*Aphis fabae*	[48]
		*Aphis craccivora*	[48]
		*Aphis gossypii*	[66]
	Cicadellidae	*Amrasca biguttula*	[46]
		*Empoasca* sp.	[47]
	Coreidae	*Anoplocnemis curvipes*	[48]
		*Clavigralla tomentosicollis*	[47]
	Delphacidae	*Sogatella furcifera*	[53]
	Miridae	*Eurystylus oldi*	[54]

Lepidoptera	Crambidae	*Cnaphalocrocis medinalis*	[50]
	Gelechiidae	*Pectinophora gossypiella*	[45,66]
		*Phthorimea operculella*	[60]
	Noctuidae	*Busseola fusca*	[74,75]
		*Helicoverpa armigera*	[45,46,50,54,66]
		*Helicoverpa zea*	[76]
		*Sesamia calamistis*	[74,75]
		*Spodoptera frugiperda*	[72,77]
	Pieridae	*Pieris rapae*	[78]
	Pyralidae	*Mussidia nigrivenella*	[49]
	Sphingidae	*Manduca sexta*	[72,79]
	Plutellidae	*Plutella xylostella*	[72]

Orthoptera	Acrididae	*Zonocerus variegatus*	[73]

Thysanoptera	Thripidae	*Megalurothrips sjostedti*	[47,48]

**Table 3:** Types of extracts evaluated and types of effects highlighted in reported studies.

Type of extract	Reported type of effect	Reference
Aqueous extract	Contact toxicity and partial chemo-sterilizing effect; delayed molting; morphogenetic lethal effect after topical application; chronic toxicity	[66]
Leaf extracts	inhibition of oviposition, anti-feedent and insecticidal effects	[58]
Dry seed powder acetone extract	Contact toxicity, ingestion toxicity	[63]
Dry seed powder and water extract	Repellency and reduced emergence	[69]
*n*-Hexane, ethyl acetate and methanol extracts from dry seed powder	Feeding deterrency	[76]
Seed oil	Anti-oviposition and ovicidal effects	[64]
Seed oil	Egg mortality	[55]
Seed oil	Contact toxicity	[72]
Seed oil	Antioviposition and ovicidal effects	[56]
Seed oil	Feeding deterrency	[73]
Seed oil	Oviposition deterrency and ovicidal effects	[49]
Seed oil	Feeding deterrency	[74]
Seed oil	Repellency and egg toxicity	[70]
Seed oil	Insect growth regulatory effect	[23]
Oil and ethanol extract	Contact toxicity	[73]
Oil and methanol extract	Reduction of amylase and LDH activities	[50]
Oil and phorbol esters	Cessation of growth and development	[72,79]
Oil and phorbol esters	Contact toxicity, ingestion toxicity, ovicidal effects and reduction of development/fertility of progeny	[54]
Phorbol esters	Stomach toxicity and antifeeding activity	[78]
Phorbol esters	Contact toxicity; reduction of food consumption, relative growth and food conversion efficiency	[77]
